# Supplementary material for: Content-rich biological network constructed by mining PubMed abstracts
Source: BMC Bioinformatics. 2004 Oct 8;5:147. doi: 10.1186/1471-2105-5-147 (PMC528731; doi:10.1186/1471-2105-5-147)
Supplement: Additional File 2 — The original results of the above study (non-essential files are deleted to keep the file size under the limit set by BMC bioinformatics). [file 1471-2105-5-147-S2.bz2 › chilibotAdditionalFile2/dip05/470ID9660793E10526/html/CCL7_CXCR3.html]

 


 **CCL7** and **CXCR3** 
  
Found 39 abstracts in PubMed, retrieved 05.  
 

 What does Google say? 
 PDF only 
| .edu only 

---

**Interactive relationship** (e.g. stimulation, inhibition, etc)

**Stimulatory relationship**- 1 LARC CCL20 whose mRNA level increased rapidly at 3 h post challenge and then gradually decreased, 2 JE CCL2, MARC  [ **CCL7** ]   **CCL7** , MIP 1gamma CCL9, monocyte chemoattractant protein MCP 5 CCL12, ELC CCL19 and BRAK CXCL14 whose mRNA levels increased with time and reached the maximum at 6 9 h post challenge, 3 LIX CXCL5, Mig CXCL9 and IP 10  [ **CXCR3** ]  CXCL10 whose mRNA levels increased gradually at least up to 12 h post challenge, and 4 SLC CCL21 whose mRNA level decreased gradually with time after challenge.  Ref: 12644322 Immunol Lett, 2003
**Neutral relationship**

**Non-interactive relationship** (e.g. studied together, co-existance, homology, etc.)

- Expression of IL 8, MCP 1, MCP 3  [ **CCL7** ] , and IP 10  [ **CXCR3** ]  was significantly higher in pouchitis than normal pouch.  Ref: 12827410 Int J Colorectal Dis< MedlineTA>Int J Colorectal Dis, 2003
- Under unstimulated conditions, no differences in the production of interleukin 8 IL 8, IFN inducible protein 10 IP 10  [ **CXCR3** ] , monokine induced by interferon gamma Mig, monocyte chemoattractant protein 1 MCP 1, and monocyte chemoattractant protein 3  [ **CCL7** ]  MCP 3  [ **CCL7** ]  were seen between untreated MS patients and controls.  Ref: 12020972 J Neuroimmunol, 2002
- From among the 13 chemokines examined, MCP 3  [ **CCL7** ] , MCP 4, RANTES, and IP 10  [ **CXCR3** ]  were detected in the disc from the idiopathic scoliosis, and MCP 3  [ **CCL7** ] , MCP 4, RANTES, IP 10  [ **CXCR3** ] , MIG, and MGSA alpha were detected in the infected or herniated discs.  Ref: 12131709 Spine, 2002
- The expression of MCP 1, MCP 3  [ **CCL7** ]  and IP 10  [ **CXCR3** ]  were significantly lower after antibiotic treatment.  Ref: 12827410 Int J Colorectal Dis< MedlineTA>Int J Colorectal Dis, 2002
- Biopsies were stained for CD68, CD3, elastase, eotaxin, IP 10  [ **CXCR3** ] , MCP 1, MCP 3  [ **CCL7** ] , and IL 8 and analyzed by NIH Image analyzer.  Ref: 12827410 Int J Colorectal Dis< MedlineTA>Int J Colorectal Dis, 2002
- In vitro addition of IFN beta1b to peripheral blood mononuclear cells PBMC cultures tended to decrease the production of IL 8, IP 10  [ **CXCR3** ] , Mig, MCP 1, and MCP 3  [ **CCL7** ] , but not of MIP 1alpha.  Ref: 12020972 J Neuroimmunol, 2002
- The RNA was reverse transcribed, and the resultant cDNA was amplified by PCR using primer pairs specific to the CXC chemokines IL 8, MGSA alpha, IP 10  [ **CXCR3** ] , MIG, the CC chemokines MCP 1, MCP 2, MCP 3  [ **CCL7** ] , MCP 4, MIP 1alpha, MIP 3alpha, RANTES, STCP 1, the C chemokine lymphotactin, and the glyceraldehyde phosphate housekeeping gene.  Ref: 12131709 Spine, 2002
